# *QuickStats:* Age-Adjusted Percentages* of Persons of All Ages Who Delayed Seeking Medical Care in the Past 12 Months Because of Worry About Cost,**^†^** by U.S. Census Region**^§^** of Residence — National Health Interview Survey,**^¶^** 2012 and 2017

**DOI:** 10.15585/mmwr.mm6818a5

**Published:** 2019-05-10

**Figure:**
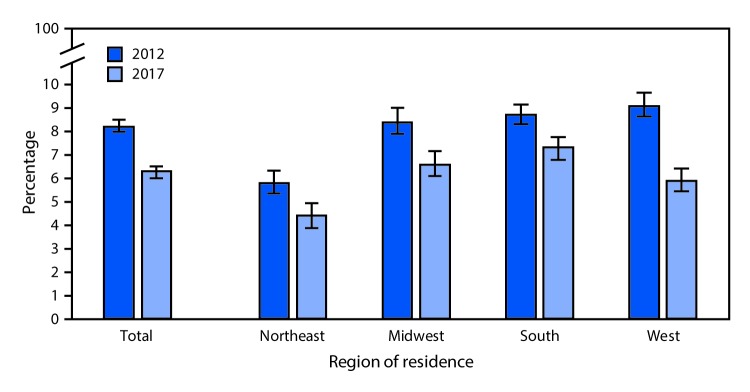
The percentage of persons of all ages who delayed seeking medical care in the past 12 months because of worry about the cost decreased from 8.2% in 2012 to 6.3% in 2017, and this pattern was consistent in each U.S. Census region of residence. Delays in seeking medical care because of worry about the cost declined from 5.8% to 4.4% in the Northeast, from 8.4% to 6.6% in the Midwest, from 8.7% to 7.3% in the South, and from 9.1% to 5.9% in the West. In both 2012 and 2017, persons of all ages living in the Northeast were the least likely to delay medical care because of worry about the cost.

**Sources:** Summary Health Statistics for the U.S. Population, National Health Interview Survey, 2012. https://www.cdc.gov/nchs/data/series/ sr_10/sr10_259.pdf.

Tables of Summary Health Statistics, 2017. https://ftp.cdc.gov/pub/Health_Statistics/NCHS/NHIS/SHS/2017_SHS_Table_P-9.pdf.

